# Exploring resting state connectivity in patients with psychotic depression

**DOI:** 10.1371/journal.pone.0209908

**Published:** 2019-01-17

**Authors:** Mardien L. Oudega, Ysbrand D. van der Werf, Annemieke Dols, Mike P. Wattjes, Frederik Barkhof, Filip Bouckaert, Mathieu Vandenbulcke, François-Laurent De Winter, Pascal Sienaert, Piet Eikelenboom, Max L. Stek, Odile A. van den Heuvel, Louise Emsell, Didi Rhebergen, Eric van Exel

**Affiliations:** 1 Department of Old age Psychiatry, GGZ inGeest Specialized Mental Health Care and Amsterdam University Medical center, location VU University medical center (VUmc), Amsterdam, the Netherlands; 2 Amsterdam Neuroscience, Vu/Vumc/UVA/AMC, Amsterdam, the Netherlands; 3 Department of Anatomy and Neurosciences, Amsterdam University Medical center, location VUmc, Amsterdam, the Netherlands; 4 Amsterdam UMC, Vrije Universiteit, Psychiatry, Amsterdam Public Health research institute, The Netherlands; 5 Department of Radiology, Amsterdam University Medical center, location VUmc, Amsterdam, the Netherlands; 6 Institutes of Neurology and Healthcare Engineering, University College London, London, United Kingdom; 7 Department of Old Age Psychiatry, University Psychiatric Center KU Leuven (Catholic University of Leuven), Leuven, Belgium; 8 KU Leuven, University Psychiatric Center KU Leuven, Academic Center for ECT and Neuromodulation (AcCENT), Kortenberg, Belgium; Shenzhen University, CHINA

## Abstract

**Background:**

Severe depression is associated with high morbidity and mortality. Neural network dysfunction may contribute to disease mechanisms underlying different clinical subtypes. Here, we apply resting-state functional magnetic resonance imaging based measures of brain connectivity to investigate network dysfunction in severely depressed in-patients with and without psychotic symptoms.

**Methods:**

A cohort study was performed at two sites. Older patients with major depressive disorder with or without psychotic symptoms were included (n = 23 at site one, n = 26 at site two). Resting state 3-Tesla functional MRI scans, with eyes closed, were obtained and Montgomery-Åsberg Depression Rating Scales were completed. We denoised data and calculated resting state networks in the two groups separately. We selected five networks of interest (1. bilateral frontoparietal, 2.left lateralized frontoparietal, 3.right lateralized frontoparietal, 4.default mode network (DMN) and 5.bilateral basal ganglia and insula network) and performed regression analyses with severity of depression, as well as presence or absence of psychotic symptoms.

**Results:**

The functional connectivity (FC) patterns did not correlate with severity of depression. Depressed patients with psychotic symptoms (n = 14, 61%) compared with patients without psychotic symptoms (n = 9, 39%) from site one showed significantly decreased FC in the right part of the bilateral frontoparietal network (p = 0.002). This result was not replicated when comparing patients with (n = 9, 35%) and without (n = 17, 65%) psychotic symptoms from site two.

**Conclusion:**

Psychotic depression may be associated with decreased FC of the frontoparietal network, which is involved in cognitive control processes, such as attention and emotion regulation. These findings suggest that FC in the frontoparietal network may be related to the subtype of depression, i.e. presence of psychotic symptoms, rather than severity of depression. Since the findings could not be replicated in the 2^nd^ sample, replication is needed before drawing definite conclusions.

## Introduction

Psychotic depression is a severe psychiatric disorder associated with high morbidity [1] and mortality [2]. Five to 20 percent of outpatients with a unipolar depression show psychotic features [3, 4, 5], characterized by mood-congruent hallucinations and/or delusions [6]. The prevalence of psychotic depression is even higher among older in-patients with rates ranging from 24 to 53%[7, 8].

There is some evidence to suggest that impaired structural and functional brain connectivity is a significant contributor to the disease mechanism of depression [9] and psychosis [10]. Network dysfunction has been studied in psychosis, including bipolar disorder and schizophrenia [11]. Results suggest an important role for the right dorsolateral prefrontal cortex (DLPFC) in forming and overcoming delusions [12, 13].

To date, only one study has focused on network dysfunction in depression with psychotic symptoms [14]. Depressed patients with psychotic symptoms showed significantly decreased functional connectivity (FC) between the hypothalamus and the subgenual anterior cingulate cortex compared to patients without psychotic symptoms[14]. The authors used seed-based resting state analyses, i.e. they estimated the networks based on a reduced set of regions, rather than studying whole-brain connectivity.

Hyett and colleagues [15] evaluated the impact of melancholic symptoms on network functioning in depressed patients, by way of independent component analysis (ICA). Their findings cannot be directly generalized to depression with psychotic symptoms, though, considering the broad array of overlapping symptomatology, including weight loss or loss of appetite, psychomotor agitation or retardation, early morning awakening, excessive guilt and worse mood in the morning [6], it has been suggested that depression with melancholic symptoms and depression with psychotic symptoms are two subtypes of depression that have a shared disease mechanism [16].

The results of Hyett and colleagues [15], using ICA and dynamic causal modelling, showed decreased FC between the right frontoparietal network and the insula in melancholic compared with non-melancholic patients. Although melancholic depression is one of the most severe subtypes of depression, Hyett did not report on a relation between severity scores and functional connectivity [15].

In this study, we evaluated resting state networks in depression with and without psychotic symptoms. Patients were included from the MODECT study (Mood Disorders in Elderly treated with Electro Convulsive Therapy), conducted at two specialized old age clinical facilities. Considering the high prevalence and admission rates of older adults with psychotic depression [7, 8], this provides an excellent opportunity to study underlying networks in psychotic depression. The aim of this study was to evaluate the relationship between resting-state network connectivity, depression symptom severity and the presence of psychotic symptoms in severe late-life depression, in order to gain insight into potential disease mechanisms underlying clinical differences in depression subtypes.

Based on the ICA study of Hyett [15], we pre-selected the following networks: frontoparietal networks, the network containing the insula together with the basal ganglia and the default mode network (DMN), all of which robustly appear as independent components across resting state studies. We hypothesized that psychotic symptoms accompanying depression are related to decreased FC of the bilateral-, left- and right frontoparietal network, DMN and the bilateral insula and basal ganglia network, and that severity of depression is not related to connectivity of these resting state networks.

## Methods

The current study is part of a two-site naturalistic, longitudinal study (MODECT: Mood Disorders in Elderly treated with Electro Convulsive Therapy)[17] including patients with severe unipolar depression according to DSM-IV-TR criteria [6] eligible for electroconvulsive therapy (ECT). Patients aged 55 years and older, referred for ECT, were recruited from tertiary psychiatric hospitals (GGZ inGeest, Amsterdam, the Netherlands (site one) and University Psychiatric Center, KU Leuven, Belgium (site two). Exclusion criteria were another major DSM-IV-TR diagnosis, such as schizophrenia, bipolar or schizoaffective disorder and a history of major neurological illness (including Parkinson’s disease, stroke and dementia). Diagnoses were made by a psychiatrist and confirmed by the Mini International Neuropsychiatric Interview (MINI) [18]. The psychiatrist evaluated the patients capacity to consent. Written informed consent was obtained from all participants. Data collection started on January 1, 2011, and finished on December 31, 2013. The local institutional boards of GGZ inGeest and the University Hospital Leuven approved the study.

### Clinical evaluations

The diagnosis of depression with or without psychotic symptoms was based on the DSM-IV-TR criteria (short MINI interview, without melancholic depression) and clinical judgment of the treating psychiatrist. Late onset depression was defined as a first episode of depression after the age of 55. Depressive symptom severity was rated with the Montgomery-Åsberg Depression Rating Scale (MADRS) [19]. Global cognitive functioning was examined by the Mini-Mental State Examination (MMSE) [20]. The MINI, MADRS and MMSE were completed by a research nurse for all patients. The duration of episode was defined as the period in months from the start of the current depressive episode until the start of ECT. Concomitant medication during ECT was accepted.

### Statistical analyses

The Statistical Package for Social Sciences software (IBM statistics 20) was used for statistical evaluation of data. Demographics and clinical characteristics of patients are reported as means with standard deviation, medians with inter-quartile range (iqr) or absolute numbers with percentage of total group. Patient sub-groups were compared using independent sample T-tests, Pearson chi-square tests or Mann-Whitney U tests, where appropriate.

### Resting state functional MRI (rsfMRI)

All patients underwent MRI scanning prior to the start of ECT, following a standard protocol. In Amsterdam, a General Electrics Signa HDxt 3.0 Tesla scanner (General Electric, Milwaukee, WI, USA) was used and in Leuven a Philips Intera 3.0 Tesla scanner (Philips, Best, The Netherlands). Patients were instructed to keep their eyes closed and not fall asleep.

The rsfMRI series in Amsterdam included a total of 202 functional images (5 minute run), acquired with an 8-channel circularized head coil using a T2*-weighted single-shot gradient echo-planar imaging sequence (repetition time = 1800ms; echo time = 35ms; 64x64 matrix; field of view = 21.1cm; flip angle = 80°) with 34 ascending slices per volume (3.3x3.3mm in-plane resolution; slice thickness = 3.0mm; inter-slice gap = 0.3mm). A coronal 3D T1-weigthed dataset was acquired for co-registration purposes (flip angle = 12°, repetition time = 7.84 milliseconds, echo time = 3.02 milliseconds; matrix 256x256, voxel size 0.94x0.94x1 mm; 180 slices).

The rsfMRI series in Leuven included a total of 250 functional images (5 minute run), acquired with an 8-channel head coil using a T2*-weighted echo-planar imaging sequence (repetition time = 1700ms; echo time = 33ms; 64x64 matrix, field of view = 230mm x 128mm x 230mm, flip angle = 90°) with 32 ascending axial slices per volume, voxel size 4 x 4 x 4 mm. A3D T1-weighthed dataset was acquired for co-registration(flip angle = 8°; repetition time = 9.6 milliseconds, echo time = 4.6 milliseconds; matrix 256x256, voxel size 0.98x0.98x1.2 mm; 182 slices).

### MRI data preprocessing steps

Data preprocessing was performed using the FSL 5.0.8 Brain Extraction Tool (BET) [21] for removal of non-brain tissue from the structural and rsfMRI scans. The first two resting state volumes of each patient time series were removed to avoid artefacts. FSL Melodic 3.14 was used for an ICA-based single-session denoising approach [22]. The following standard processing steps were applied: high-pass filtering (100s), motion correction with MCFLIRT [23] for removal of head motion, voxel-wise demeaning, normalization of the voxel-wise variance, and spatial smoothing using a Gaussian kernel of FWHM = 5.0 mm with high-pass filtering. The preprocessed data were then linearly registered to the structural image using FLIRT with optimization and registered to MNI space using 12 and 7 degrees of freedom, respectively. An automated component classification method, called FIX 1.061 (http://fsl.fmrib.ox.ac.uk/fsl/fslwiki/FIX), was then used to classify and to regress out the noise time series from the data. This method regresses out unique variance related to the noise components and motion confounds from the preprocessed datasets. This resulted in cleaned, EPI time-series for each patient.

### Independent component analyses

Multi-session temporal concatenation ICA was used to analyze group ICAs with dimensionality set to 30 (indicating the number of networks to be extracted in the analysis). Due to the use of different scanners and imaging protocols at both sites, the datasets were analyzed separately. The set of spatial maps from the group-average analysis was used to generate subject-specific versions of the spatial maps, and associated timeseries, using dual regression [24]. First, for each subject, the group-average set of spatial maps is regressed (as spatial regressors in a multiple regression) into the subject's 4D space-time dataset. This results in a set of subject-specific timeseries, one per group-level spatial map. Next, those timeseries are regressed (as temporal regressors, again in a multiple regression) into the same 4D dataset, resulting in a set of subject-specific spatial maps, one per group-level spatial map. We then tested for [group differences, etc.] using FSL's randomize permutation-testing tool. Randomize was performed with default settings; i.e. 5000 segmentations. We then used threshold free cluster enhancement (TFCE) with voxel-based thresholding, corrected for multiple comparisons by using the null distribution of the max voxelwise test statistic [25].

Dual regression analyses [24] followed by randomize [25] for the statistical evaluation were used with dichotomous variables for the group comparisons and demeaned continuous variables of the MADRS scores (depression severity) for the correlation analysis. To remove variation associated with age in the analyses, we adjusted the general linear models for age and gender during the dual regression processing. Masks were created from main effects of network across participants and then binarized at z>3 for both centers separately. Masks were made from the 1.bilateral-, 2.left- and 3.right frontoparietal network, 4.DMN and 5.bilateral insula and basal ganglia network produced by the group ICAs with Z = 3.0. All individual analyses were cluster corrected using threshold free cluster enhancement and we performed a further correction for multiple comparisons so that we divided the corrected p value of 0.05 by a factor 10 (five networks x 2 for two-tailed testing) leaving a significance threshold of p<0.005.

## Results

**Fig 1:**
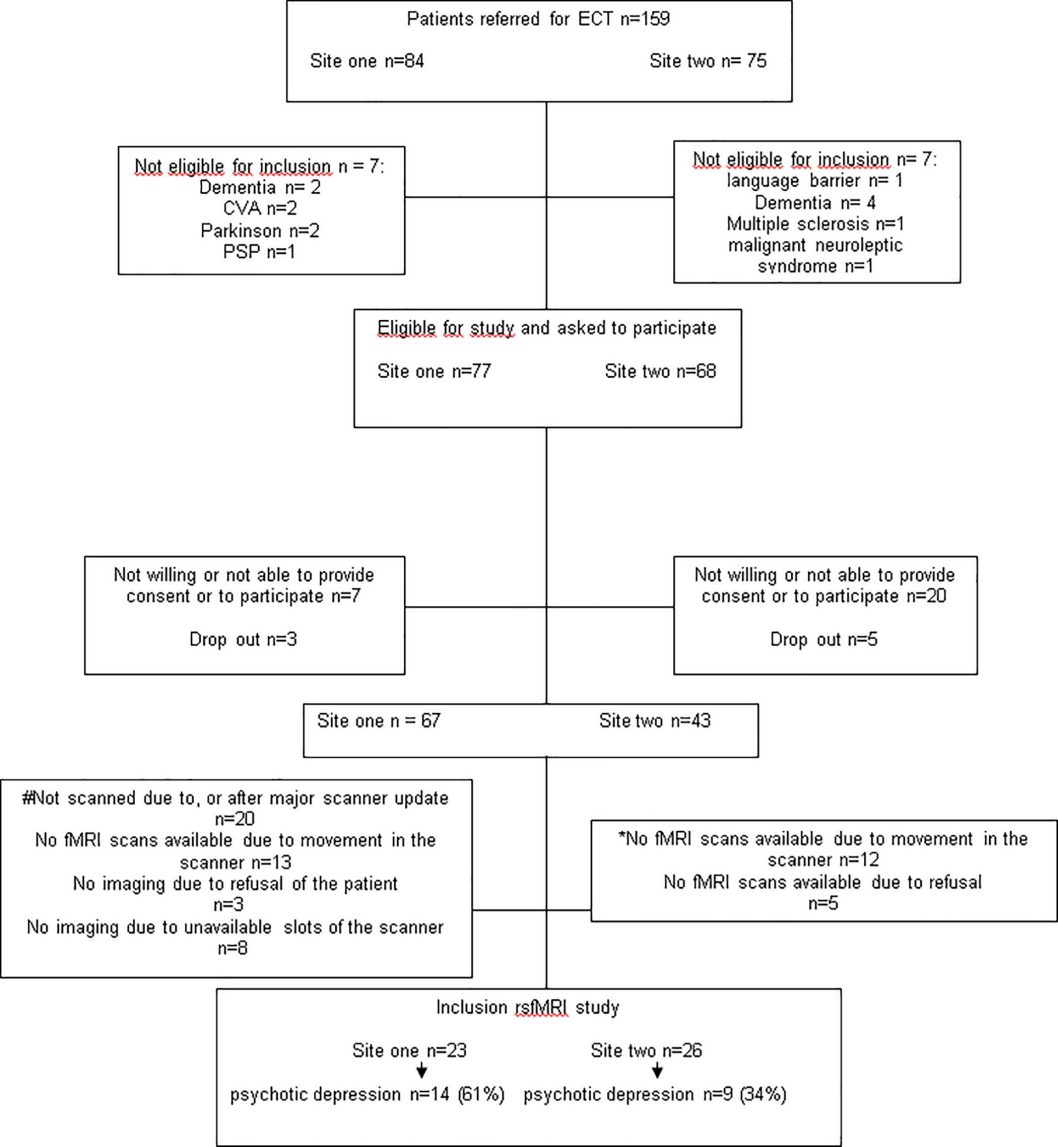
Flow chart. At site one, ten patients did not have a scan due to a major scanner update and ten patients were excluded from analyses because they were scanned after a major scanner upgrade. Thirteen patients could not finish the fMRI protocol adequately, due to movement in the scanner and no imaging data could be obtained from 11 patients due to refusal (3 patients) or unavailable slots of the MRI scanner (8 patients). Nine patients out of these 24 patients were diagnosed with a psychotic depression. In the end, 23 of the 67 recruited patients were included in the analyses of site one. *At site two, 12 patients could not finish the fMRI protocol due to movement in the scanner and five patients refused an fMRI scan. Seven out of these 17 patients without a rsfMRI scan were diagnosed with psychotic depression at site two. In the end, 26 of the 43 recruited patients were included in the analyses of site two.

Fig 1 shows a flow chart of 159 patients referred for ECT and asked to participate in the current study. Twenty-three patients were included in the analyses of site one and 26 patients were included in the analyses of site two (see flow diagram Fig 1).

### Characteristics

There were no significant differences between site one and site two with respect to age, gender, presence of psychotic symptoms, age at onset of first depression, depression symptom severity (total MADRS score), cognitive functioning (total MMSE score), duration of episode (Table 1). At site one five depressed patients with psychotic symptoms used concomitant medication during scanning (one patient used antipsychotic medication, three patients used antidepressant medication and one patient used a mood stabilizer). Two depressed patients without psychotic symptoms used antidepressant medication during scanning. At site two one depressed patients with psychotic symptoms used medication and eight depressed patients without psychotic symptoms used medication. The type of medication was not registered at site two.

**Table 1:** Demographics and clinical characteristics of patients included in the study (n = 49), site 1 (n = 23) and site 2 (n = 26).

Demographics	Site 1	Site 2	P*	Total patients
Number of patients included (n)	23	26		49
Age (mean, sd)	68.7 ± 8.3	72.0 ± 7.4	0.15	71.0 ± 7.6
Female (n, %)	16 (70%)	17 (65%)	0.76	33 (67%)
Depression with psychotic symptoms (n, %)	14 (61%)	9 (35%)	0.07	23 (47%)
Late onset (n, %)	9 (39%)	15 (58%)	0.20	24 (49%)
MADRS (mean, sd)	32.9 ± 11.9	33.8 ± 6.7	0.74	34.6 ± 8.6
MMSE (median, iqr)	26.5 iqr 9	26.0 iqr 4	0.80	26.0 iqr 6
Duration of episode (median, iqr)	8.5 iqr 20.0	6.0 iqr 5.0	0.28	6.5 ± 10

* p value of difference between site 1 and site 2.

MADRS = the Montgomery-Åsberg Depression Rating Scale, MMSE = Mini-Mental State Examination, iqr = inter quartile range.

### Resting state networks

**Fig 2:**
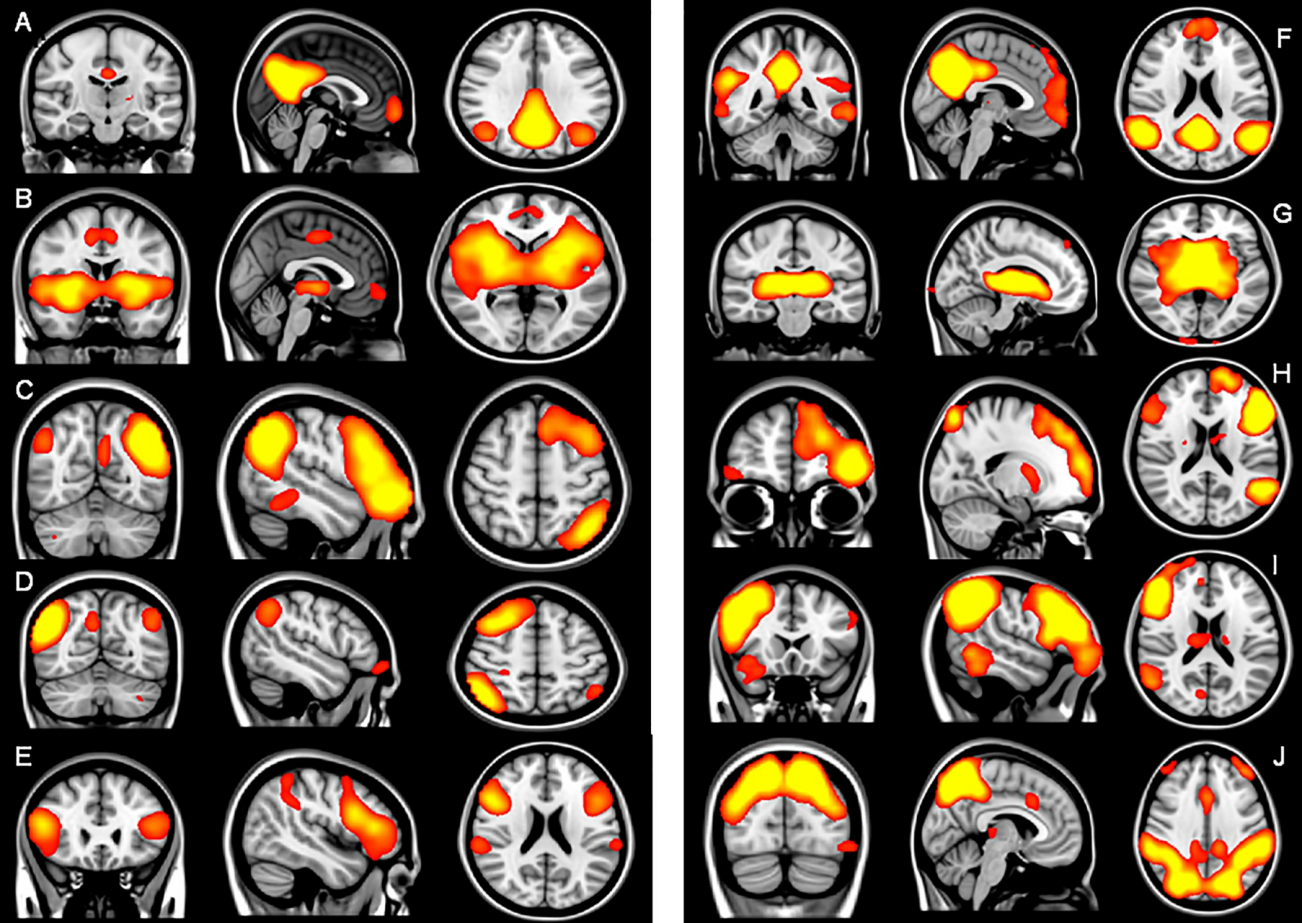
Selected networks in site one (A-E) and site two (F-J). Coronal, sagittal and axial view of resting patterns of the DMN (A x = 1, y = -17, z = 30 and F x = -3, y = -45, z = 21), Basal ganglia and insula (B x = -1, y = 1, z = 2 and G x = -11, y = -23, z = 3), frontoparietal left (C x = -49, y = -63, z = 52 and H x = -18, y = 55, z = 19), frontoparietal right (D x = -49, y = -63, z = 52 and I x = 55, y = 19, z = 19) and frontoparietal bilateral (E x = 46, y = 25, z = 21 and J x = -5, y = -81, z = 35). Images are thresholded at z>3.0.

Melodic multi-session temporal concatenation with automatically set threshold identified 46 and 59 fragmented components of networks, respectively, at site one and site two. After visual inspection multi-session temporal concatenation ICA was set at 30 resting state networks. Among these 30 networks the DMN, insula and basal ganglia, medial visual, separate unilateral (right and left) and bilateral frontoparietal, motor cortex, auditory, sensorimotor, executive control and cerebellum were consistently found, corresponding to those reported in previous studies [26]. Among the selected 30 networks, 28 were labeled as the same based on visual inspection and classification by agreement between two raters (MO and YvdW). We selected the a priori defined networks-of-interest by visual inspection on the basis of the networks as they followed from the group ICA: 1.bilateral-, 2.left- and 3.right frontoparietal network, 4. DMN and 5. bilateral insula and basal ganglia (Fig 2). Neither gender or patient age were associated with functional connectivity in any of the specified networks at either site.

### Correlation with severity of depression

The mean MADRS score was 32.9 (sd ± 11.9) and 33.8 (sd ± 6.7) for site one and site two, respectively. The dual regression analyses showed no significant associations between severity of depression (total MADRS score) and FC of the bilateral-, left- and right frontoparietal network, DMN and bilateral insula and basal ganglia network at either site (all p>0.005).

### Relation with presence of psychotic symptoms

**Fig 3:**
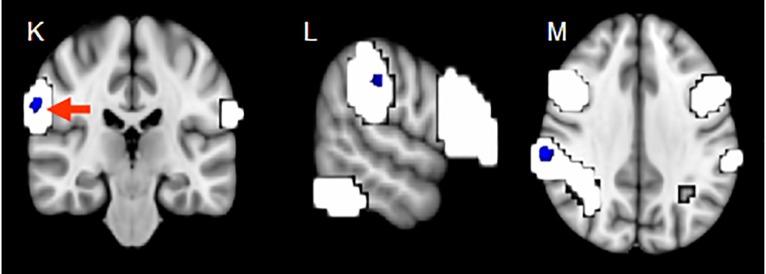
Decreased functional connectivity in the right part of the frontoparietal bilateral network of depressed patients with psychotic symptoms compared with patients without psychotic symptoms at site one, in blue (x = 60, y = -25, z = 33, p = 0.002; for visualization purposes shown here cluster-corrected p<0.05), overlaid on the frontoparietal network (white, thresholded at z>3.0).

At site one, 14 patients (61%) out of 23 depressed patients were diagnosed with a depression with psychotic symptoms. These patients showed significantly lower FC in the right part of the bilateral frontoparietal network, compared with the depressed patients without psychotic symptoms (p = 0.002, Tmax = 6.72, cluster size = 12 and the coordinates in MNI space are: x = 60, y = -25, z = 33) (see Fig 3).

At site two, nine patients out of 26 (35%) were diagnosed with depression with psychotic symptoms. No significant differences were observed in FC of the bilateral-, left- and right frontoparietal network, DMN and (bilateral basal ganglia and insula network) in the depressed patients with psychotic symptoms compared with the depressed patients without psychotic symptoms (all p>0.005).

## Discussion

To our knowledge this is the first study evaluating FC of resting state networks using ICA with five networks of interest in patients with severe depression with and without psychotic symptoms who were eligible for ECT and admitted to inpatient clinics.

Resting state FC was not associated with depression symptom severity. These findings are in line with a previous study [27] and may be explained by lack of variation in symptom severity of the studied patient samples, or FC may not be state-dependent, but dependent on the subtype of depression.

Psychotic depression, compared with non-psychotic depression, was associated with decreased FC in the right part of the bilateral frontoparietal network, at site one. This result was not replicated at site two.

The frontoparietal network is involved in cognitive control processes, such as attention and emotion regulation [28]. Previous resting state studies in patients with psychosis, unrelated to depression, also showed decreased FC of the frontoparietal network [29]. Moreover, Corlett et al. 2007, 2010 [12, 13] showed a pertinent role for the right DLPFC in forming and overcoming delusions in patients with psychosis and schizophrenia. The DLPFC plays an important role in the frontoparietal network. This may suggest a similar pathophysiological mechanism across mental disorders with psychotic symptoms, possibly related to diminished cognitive control and as a result, increased vulnerability to develop psychotic symptoms. This hypothesis fits with the conclusion of a large review of resting-state studies in patients at risk of developing psychosis [30]. In addition, it has been suggested that decreased FC of the right frontoparietal network is related to the vulnerability for relapse in patients with psychotic depression [31].

Caldiero et al. [16] suggested a shared disease mechanism between melancholic depression and depression with psychotic symptoms, as these profiles both present with severe weight loss or loss of appetite, psychomotor agitation or retardation, early morning awakening, excessive guilt, and worse mood in the morning [6]. Hyett and colleagues [15] analyzed resting-state FC in patients from an outpatient clinic and demonstrated that decreased FC between the right frontoparietal network and insula was associated with melancholic depression, as compared to non-melancholic depression. Our findings show decreased FC in the right part of the bilateral frontoparietal network in psychotic versus non-psychotic depressed elderly. This may suggest a shared disease mechanism between melancholic and psychotic depression. Hypoconnectivity of the frontoparietal network was also shown in a large meta-analysis of seed-based resting state FC analyses in heterogeneous groups of patients with major depressive disorder compared with healthy controls [9].

Although the results from the analyses at site one are consistent with findings from Hyett and colleagues [15], in site two we did not find a similar association between resting state FC and the presence of psychotic symptoms. This might be explained by differences in scanner protocol and limited statistical power. Nine patients (35%) at site two with psychotic symptoms were able to complete the fMRI scanner protocol compared with 14 (61%) at site one. Differences in scanner protocol may have influenced the number of included patients with psychotic symptoms at site two as the protocol required a longer scanning time (due to requirements for another study) and patients with psychotic features may not have been able to complete the scan protocol.

### Strengths and limitations

To our knowledge, this is the first resting state fMRI study evaluating FC in depression with and without psychotic symptoms in patients who were referred for ECT and admitted to inpatient clinics. A particular strength of the study is that it enrolled a naturalistic population of severely depressed older adults under clinical care. Moreover, we analyzed two independent data sets with similar demographics and clinical/imaging protocols; the different results are interesting because they emphasize the importance of the power needed to detect differences, and the reproducibility across sites. Another important strength is the inclusion of patients with depression with psychotic symptoms. However, since the study was parallel but subordinate to patient care, some patients needed ECT before inclusion could be completed, or patients were not willing to participate (Fig 1). Also, the diagnosis of MDD with or without psychosis was based on a one-month diagnosis. We unfortunately did not have access to information about a lifetime history of psychosis of the patients so we cannot rule out the influence of trait vulnerability in our patients. Moreover, we were not able to add a healthy control group to our analyses, which would have improved our study.

Furthermore, at site two the resting state scan was acquired at the end of the total scan protocol so more people didn’t complete the resting state scan (at the end) than the T1 scan (at the beginning), possibly explaining why fewer psychotic patients completed the scan protocol. As a result, we have included a relatively small sample of patients with psychotic symptoms and may have underestimated the group differences.

Despite large overlap in the networks derived from both sites, it is possible that comparability of the components between sites was not always perfect and this may have added to a reduced sensitivity of our analyses.

The use of antidepressant or antipsychotic medication during scanning is another important confounder of our results. The analyses of both patient samples were not corrected for use of medication.

Finally, we did not obtain DSM-IV-TR [6] diagnoses of melancholic depression. This prevented a comparison between psychotic and melancholic symptoms and may represent a comparison between patients with psychotic depression versus a more heterogeneous patient group. It is possible that this caveat in our study is biased towards an underestimation of the true difference in FC of the frontoparietal network in those with psychotic depression compared to those without psychotic depression.

### Conclusion

Psychotic features, not the severity of depression, were associated with FC of the resting-state frontoparietal network in the patient sample of site one. This result was not observed in the patient sample of site two. The findings of site one suggest that FC in the frontoparietal network may be related to subtype of depression, i.e. presence of psychotic symptoms, rather than severity of depression, which is consistent with previous findings [26]. However, replication is needed to confirm this conclusion.
